# Supplementary material for: Buildup of a highly twisted magnetic flux rope during a solar eruption
Source: Nat Commun. 2017 Nov 6;8:1330. doi: 10.1038/s41467-017-01207-x (PMC5673903; doi:10.1038/s41467-017-01207-x)
Supplement: Supplementary file 1 — Supplementary Information [file 41467_2017_1207_MOESM1_ESM.pdf]

Supplementary Table 1: Comparison of magnetic flux between the solar magnetic flux rope (MFR) and its interplanetary counterpart, i.e., magnetic cloud (MC)

	Solar MFR	Interplanetary MC		
		Gold-Hoyle	Lundquist	Grad-Shafranov
Φ_p (10^{21} Mx)	$2.058^{+0.023}_{-0.031}$	$4.897^{+2.065}_{-1.942}$	5.937 ± 1.318	4.509 ± 1.002
Φ_t (10^{21} Mx)	$0.503^{+0.003}_{-0.004}$	$1.226^{+0.535}_{-0.467}$	0.997	0.919
$\Phi_\mathrm{p}/\Phi_\mathrm{t}$	$4.09^{+0.01}_{-0.02}$	3.99 ± 3.43	5.95 ± 1.32	4.91 ± 1.09

For the solar MFR, Φ_p and Φ_t are averaged over 15:30–16:00 UT.

$\Phi_\mathrm{p}/\Phi_\mathrm{t}$ estimates the average twist number within the MFR[1], which is equivalent to the twist number in a cylindrical MFR with uniform twist, i.e., the Gold-Hoyle model.

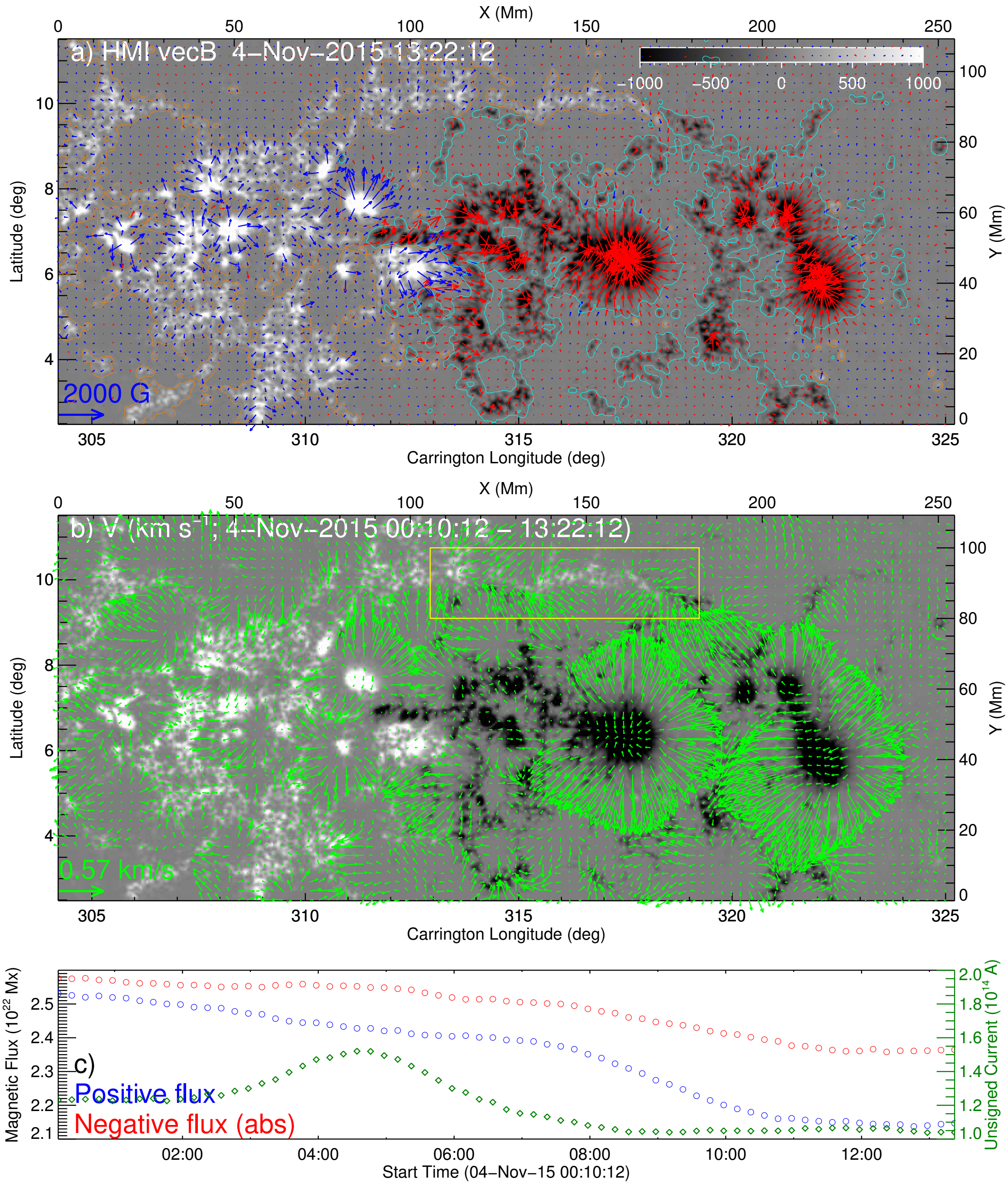

Supplementary Figure 1: Magnetic configuration of the active region of interest, NOAA 12443. a) Vector magnetogram immediately before the flare. Transverse component of the photospheric magnetic field is indicated by blue (red) arrows originating from positive (negative) polarity. Vertical component B_z is saturated at ±1000 G (see the color bar); orange (cyan) contours indicate B_z of 50 (-50) G. b) Flow map averaged over a 13-hr time period prior to the flare. The yellow rectangle marks a region characterized by converging flows, where a filament is located along the polarity inversion line (Figure 1). Maximum flow speed is 0.57 km s^{-1} as denoted by the arrow at the lower left corner. c) Variation of the integrated positive and negative magnetic fluxes and unsigned electric current (dark green) through the active region during the same 13-hr time period as annotated in the (b).

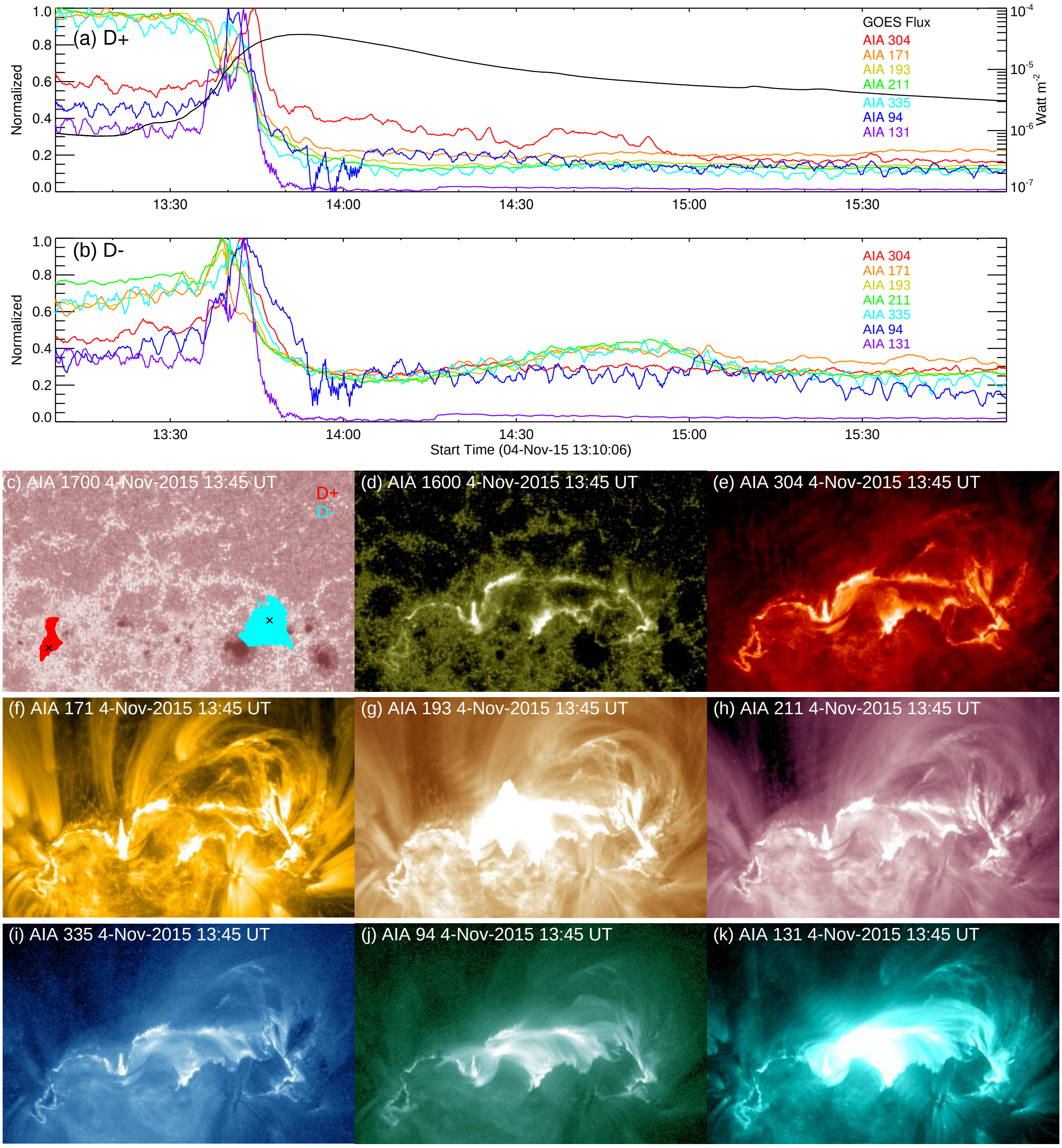

Supplementary Figure 2: Coronal dimmings. The dimmings at the far ends of flare ribbons are visible in all of 7 EUV passbands of Atmospheric Imaging Assembly (AIA), and the bright rings enclosing the dimming areas are also visible in all the EUV passbands as well as in UV 1600 Å. Panels (a and b) show the brightness of the most dimmed pixels in EUV (normalized for each individual passband) in the maximum dimming regions as indicated in (c), which are labeled 'D+' and 'D-', respectively, according to the associated magnetic polarity. Panel (a) is superimposed by 0.1–0.8 nm soft X-ray flux observed by the Geostationary Operational Environmental Satellite (GOES). Two crosses in (c) mark the most dimmed pixes when dimming was first identified and before this time data are simply taken from these two pixels.

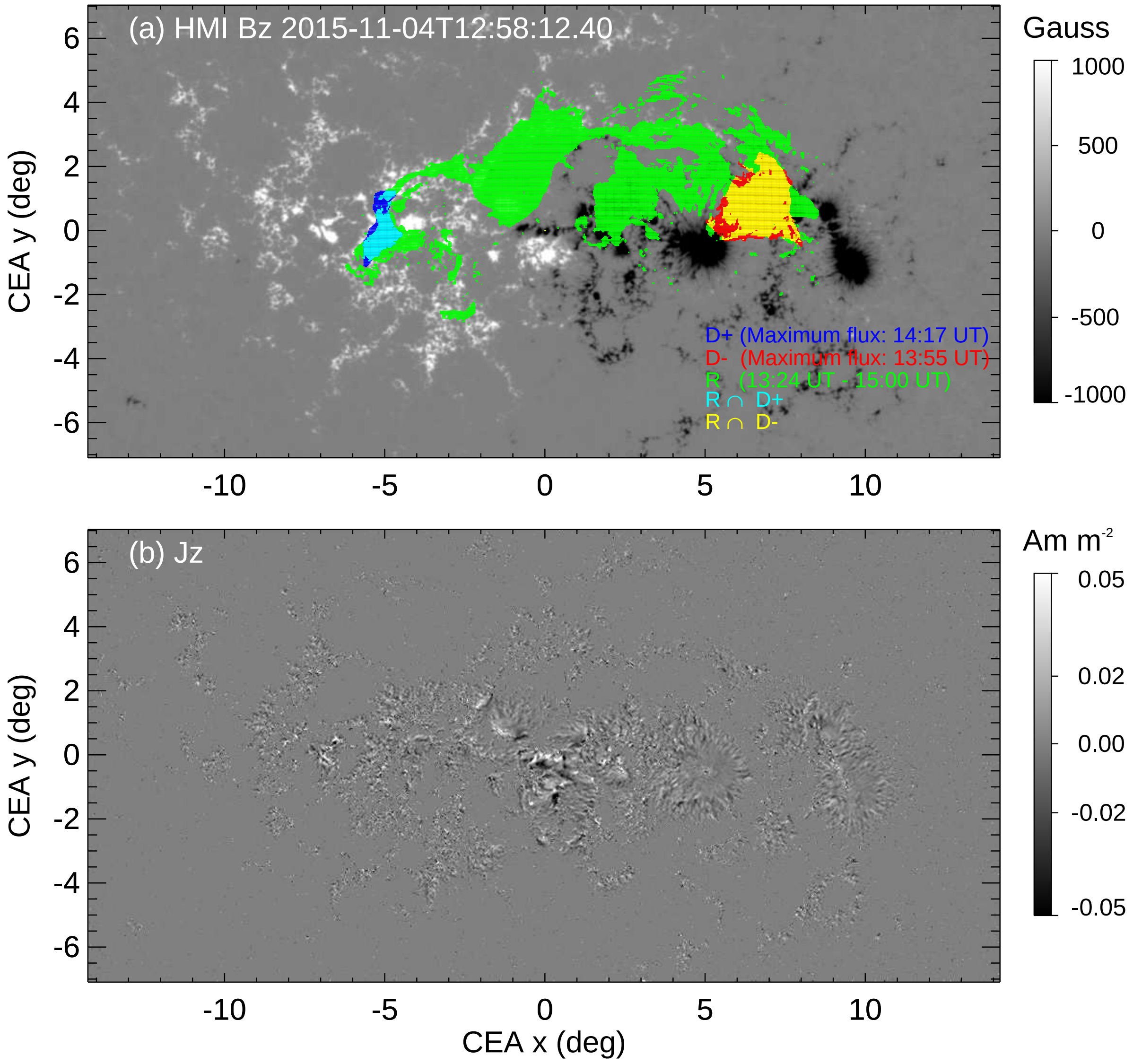

Supplementary Figure 3: Flare ribbons (including the bright rings) and coronal dimmings in relation to the photospheric magnetic field. Panel (a) shows the accumulative ribbon-swept areas in 1600 Å and maximum dimming areas in 304 Å as projected upon a pre-flare map of B_z. Note that the dimmings of opposite polarity (D+ and D-) reached maximum at different times. The ribbon-swept area overlapped with D+ (D-) is shown in yellow (cyan). Panel (b) shows the map of current density J_z in units of Am m^{-2} derived from the same vector magnetogram as (a).

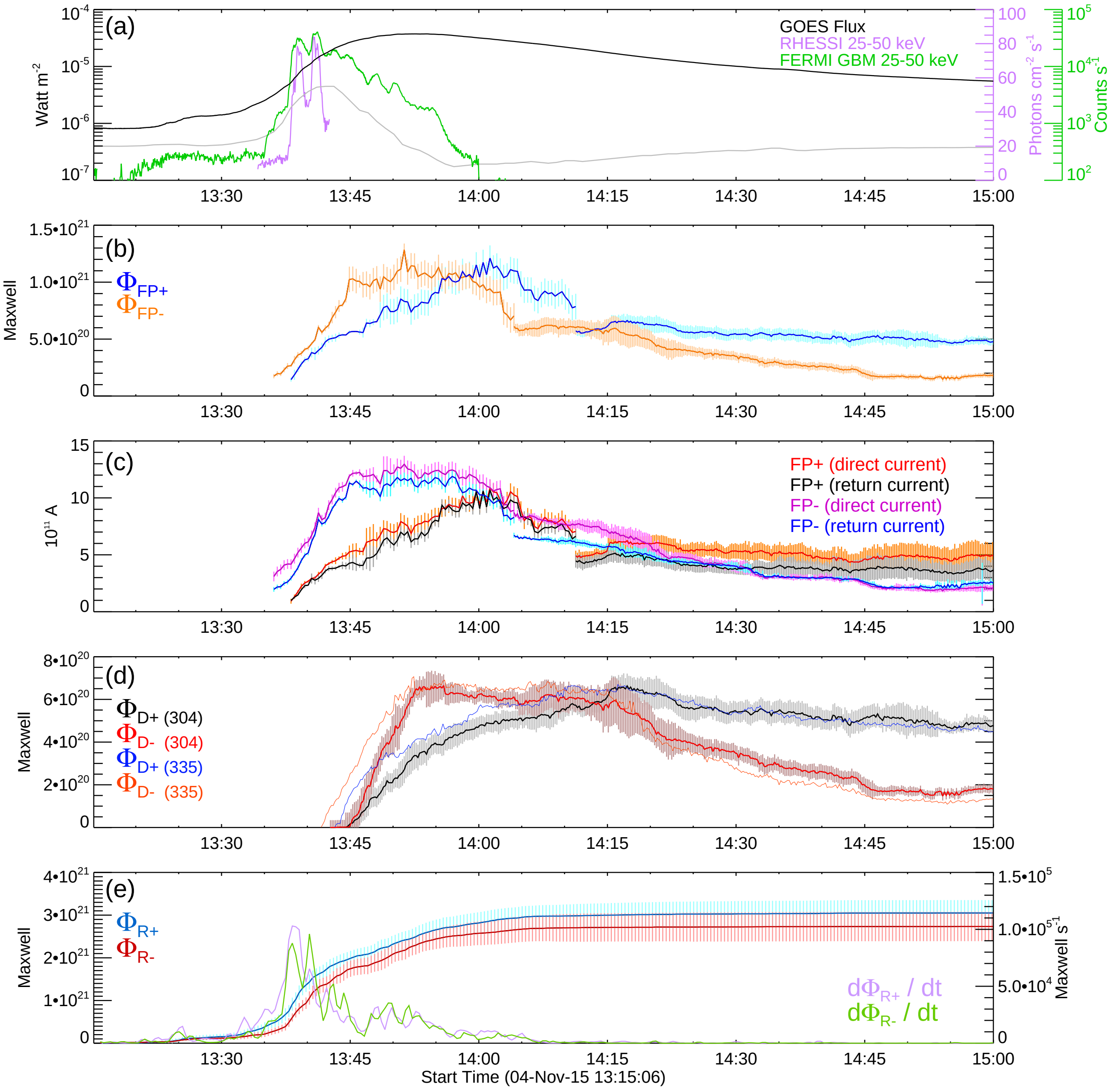

Supplementary Figure 4: Temporal evolution of magnetic flux through various features. a) 0.1–0.8 nm soft X-ray flux observed by the Geostationary Operational Environmental Satellite (GOES), its time derivative in an arbitrary unit (gray), 25–50 keV hard X-ray (HXR) photon count rate observed by the Gamma-ray Burst Monitor (GBM) onboard the Fermi Gamma-ray Space Telescope, and 25–50 keV HXR photon flux observed by the Reuven Ramaty High-Energy Solar Spectroscopic Imager (RHESSI). b) Magnetic flux through the foopoint areas FP+ and FP- of the magnetic flux rope. Note before about 14:10 UT FP+ and FP- are identified with the areas enclosed by the bright rings, but afterwards with the dimming areas. c) Direct current (J_z in the same direction as B_z) and return current within FP+ and FP-. d) Magnetic flux through the dimming areas D+ and D- as identified in 304 and 335 Å. e) Magnetic flux through the accumulative ribbon-swept areas as well as the newly brightened areas, the latter of which is the time derivative of the former. Error bars in Panels (b–e) are given by varying the detection threshold of the corresponding features (Methods).

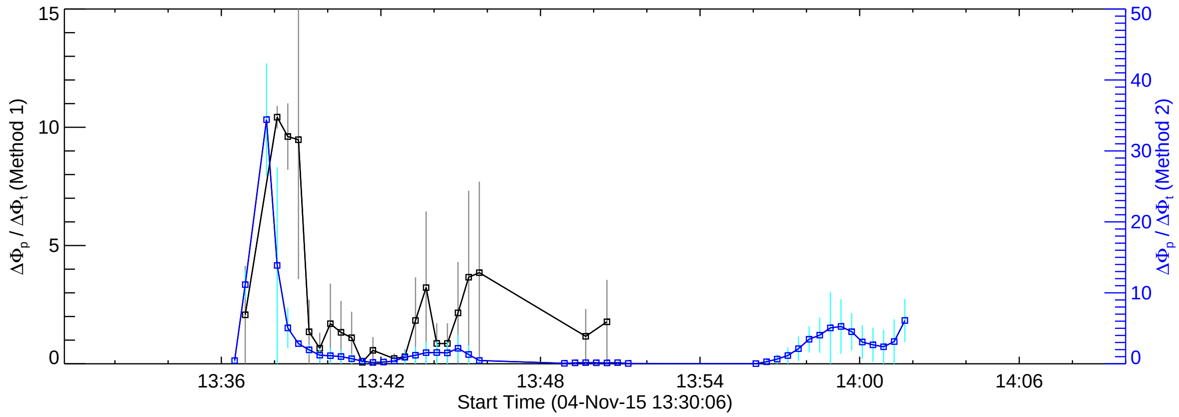

Supplementary Figure 5: Two different approaches to calculate $\Delta\Phi_{\rm p}(t)/\Delta\Phi_{\rm t}(t)$. Error bars are derived following the error propagation rules, given the uncertainties of $\Phi_{\rm p}$ and $\Phi_{\rm t}$. (Methods).

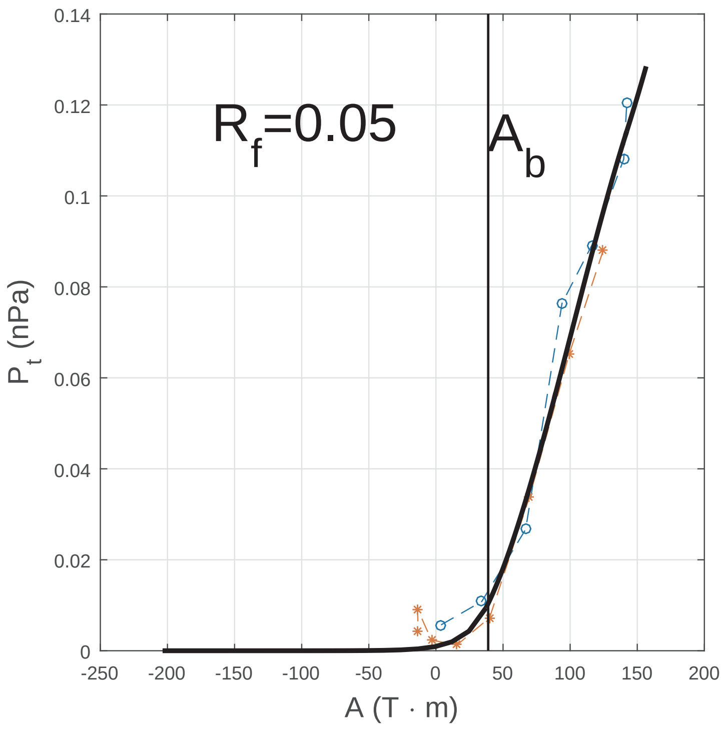

Supplementary Figure 6: A functional fitting $P_t(A)$ (thick black line) of the measurements (symbols) along the Advanced Composition Explorer spacecraft path of the magnetic cloud on 2015 November 7. The fitting residue R_f (annotated) and the boundary $A_b = 38.9$ T·m (vertical line) are defined in ref[2]. At the flux rope center, $A = 160.4$ T·m (labeled A_0 in Figure 4c).

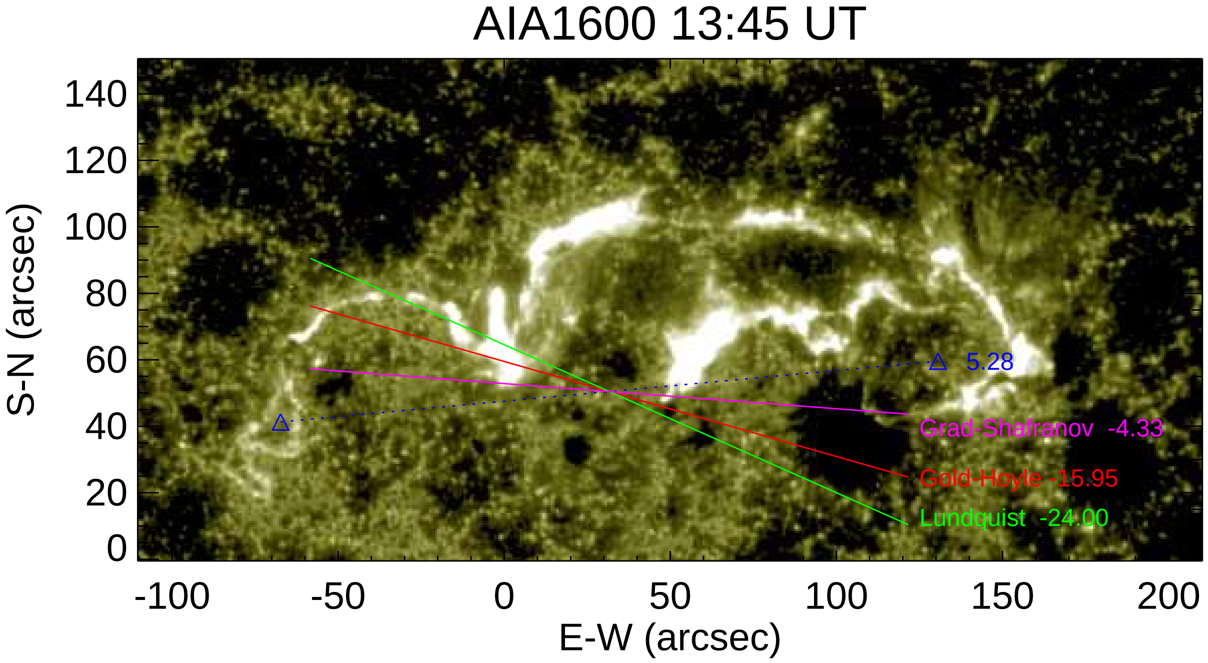

Supplementary Figure 7: Magnetic cloud (MC) axis orientations projected on the solar surface. The blue dotted line connected the centers (triangle) of FP+ and FP-, which can be considered as a proxy of the flux-rope orientation on the Sun. The magenta, red and green lines indicate the MC axis orientations given by the Grad-Shafranov reconstruction, force-free fittings based on the Gold-Hoyle and Lundquist models, respectively. The tilt angles with respect to the east-west direction (counter-clockwise positive) are given in units of degree.

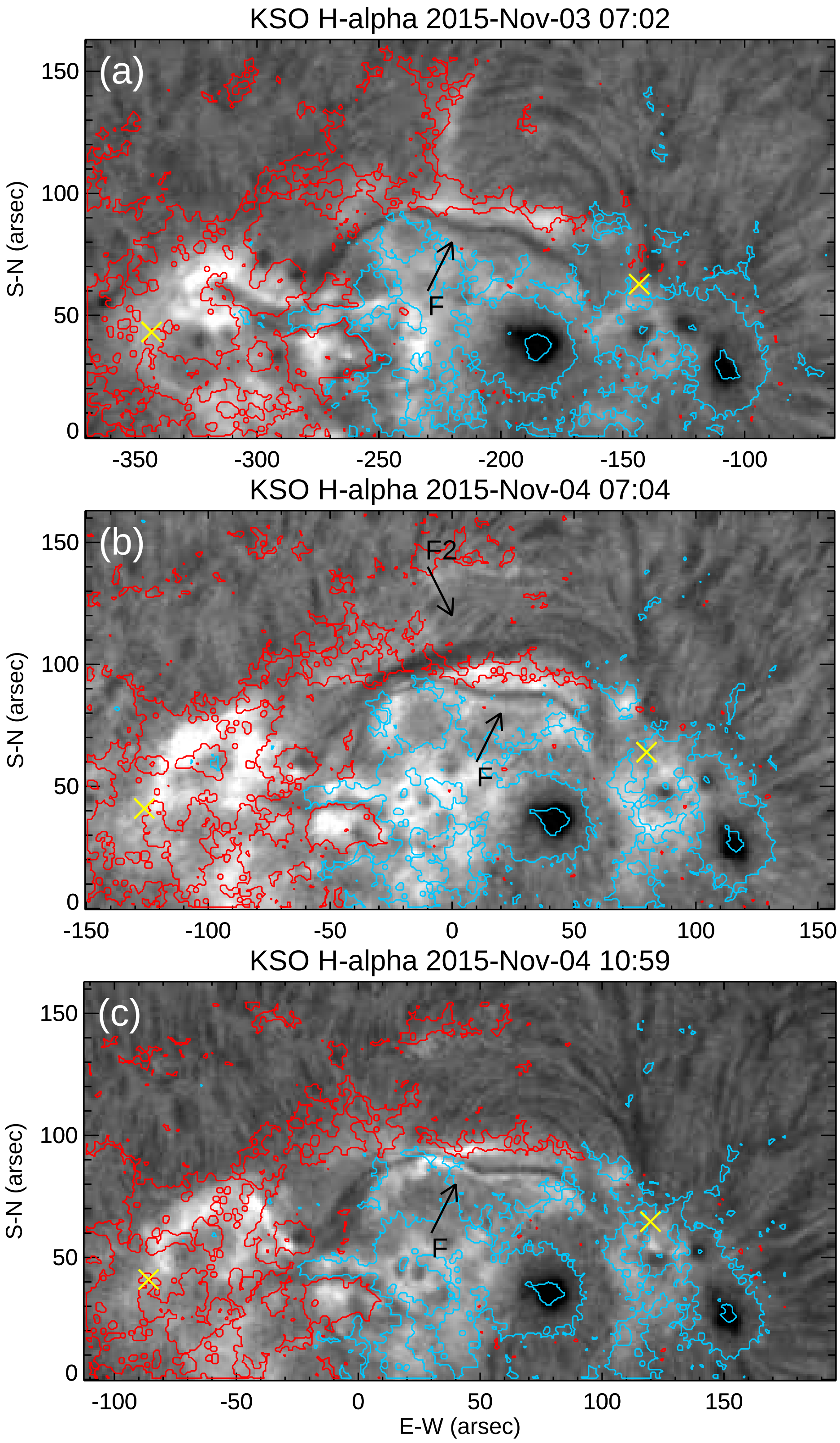

Supplementary Figure 8: The filament F as observed in Hα by the Kanzelhöhe Solar Observatory (KSO). The daily observing time at KSO is 07–13 UT. Superimposed are the contours of line-of-sight magnetic field at ±100 and ±1000 Gauss, with red (blue) indicating positive (negative) polarity. The initial footpoint brightenings of the eruptive flux rope are marked by two yellow crosses, differentially rotated to the time of each Hα image.

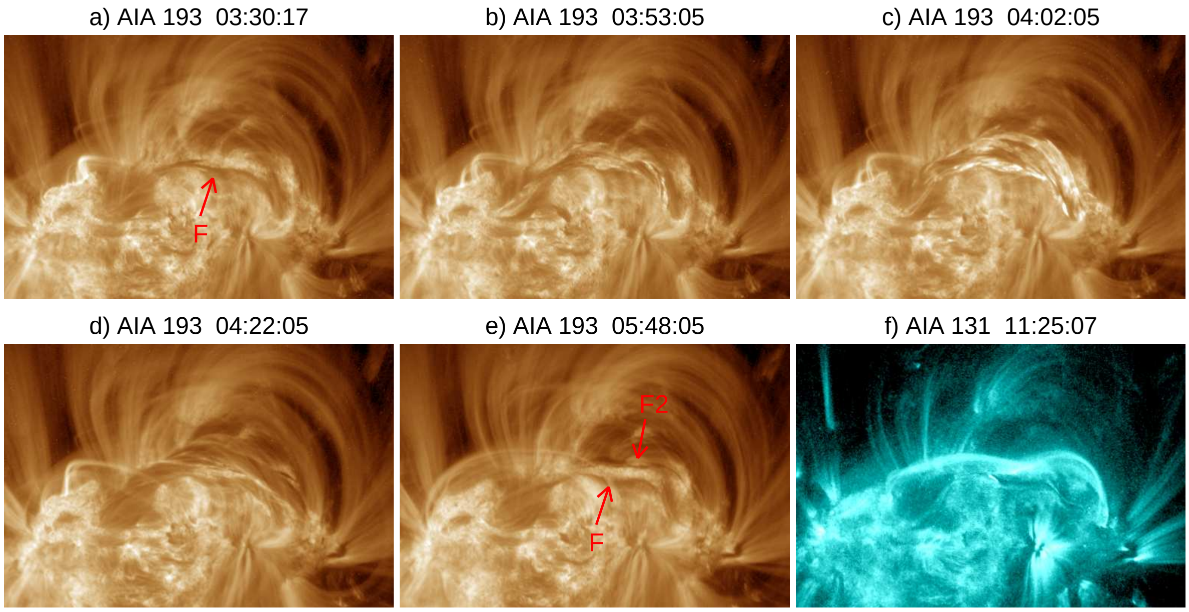

Supplementary Figure 9: Snapshots of Atmospheric Imaging Assembly (AIA) images showing the separation of the filament F into two branches. The process was associated with a C1.4 flare, staring at 03:53 UT and peaking at 03:59 UT on 2015 November 4. During the C-flare, F rose slightly and was heated, exhibiting apparently entangled threads in AIA 193 Å (Panels (c–d)). From about 04:30 UT, F2 and F started to separate due probably to the draining of filament material, and became clearly separated by about 05:00 UT. Afterwards F2 faded and completely disappeared by about 11:00 UT, and was temporarily replaced by a hot loop in 131 Å (Panel (f)).

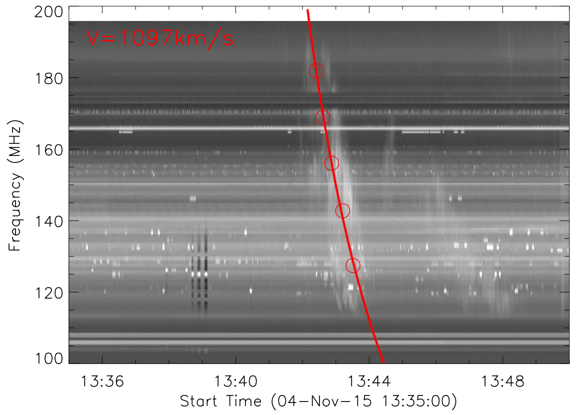

(a)

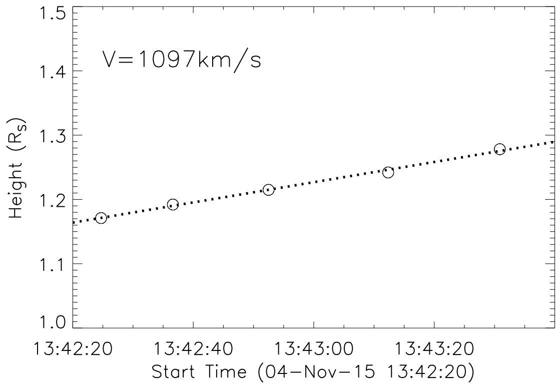

(b)

Supplementary Figure 10: Radio Type II burst. Panel (a) shows a dynamic spectrum recorded by the Bruny Island Radio Specrometer. The circles mark the selected points along the drifting feature, which is translated to a linear height-time profile with the Newkirk density model in Panel (b).

Supplementary Note 1

Signature of a preexisting MFR

The filament F was persistent prior to the eruption, but temporarily exhibited a double-decker configuration[3] during 05:00–11:00 UT on 4 November 2015. In Hα (Supplementary Figure 8) one can see that F was strictly aligned along the PIL, while another filament (labeled F2) with similar orientation was projected slightly to the north of the PIL. Most likely this is because F2 was suspended higher in the corona than F. This configuration was formed in the wake of a C1.4 flare, during which F rose slightly and was heated, exhibiting apparently braided threads in AIA 193 Å (Supplementary Figure 9 (c–d)). F started to bifurcate from about 04:30 UT and became clearly separated at 05:48 UT (Supplementary Figure 9e), due probably to the draining of filament material. Note F still retained the original shape, but F2 faded and completely disappeared by 11:00 UT. During 11:00–11:30 UT F2 appeared to be replaced by a hot loop only visible in 94 and 131 Å (Supplementary Figure 9f), which shared the similar shape as F2 and similar footpoint locations as F. Overall the filament's feet were always located in the neighborhood of the PIL (Supplementary Figure 8), and relatively fixed even during the filament activity leading to the double-decker configuration (Supplementary Figure 9). There was no sign of high twist and the hot loop in 131 Å (Supplementary Figure 9f) appeared to be twisted by about one turn at most. The loop highlighted where a possible MFR was anchored.

Two possible field configurations have been proposed for a double-decker filament, i.e., i) a double MFR and ii) a single MFR situated above a sheared arcade[3,4]. Numerical experiments have demonstrated that a partial eruption is possible with the upper branch becoming unstable and the lower one remaining in place[4]. Thus, F2 is most likely associated with a pre-existing flux rope, but its transient presence makes it unclear whether the rope was still present at the eruption onset. It is also unclear whether F was associated with a flux rope or a sheared arcade. In any case, the filament field must have undergone significant changes, i.e., magnetic reconnection with the surrounding sheared field, before being incorporated into the full-fledged MFR, because the MFR's two feet were distinct from the filament's, especially for FP+ (Figure 1 and Supplementary Figure 8), as a result of the extension of flare ribbons deep into the flux concentrations.

Interaction of the MFR with the ambient field

The interaction of the MFR with the ambient flux is evidenced by the rapid reduction of Φ_{D-} (Supplementary Figure 4), when the western bright ring shrank and encroached into D- during the flare gradual phase (see Supplementary Movie 2). This is consistent with the WIND

observation of suprathermal electron beams, which were dominated by an unidirectional flow with pitch angle < 45 deg (Figure 4), instead of counterstreaming flows often associated with interplanetary CMEs (ICMEs)[5]. Further, the ICME's interval as determined by several indicators[5] including elevated ratio of O^{7+}/O^{6+}, enhanced $\langle Q \rangle_{\mathrm{Fe}}$, and depressed T_p/T_{exp} and plasma β extends several hours before and after the MC boundary, which is identified mainly by the smooth rotation of magnetic field. This may also result from interactions between the MFR and the ambient field.

EUV Wavefront and Shock

An EUV wavefront appeared ahead of the MFR at 13:37 UT (Figure 5), as soon as the MFR's feet began to take shape and the fork-like feature was seen in the AIA's hot passbands like 131 Å. The wavefront in the 'cool' passbands like 171 and 211 Å was visible till about 13:43 UT. Meantime, a Type II radio burst drifted from 190 to 115 MHz during 13:42–13:44 UT, which gives an average shock speed of 1100 km s^{-1} with the two-fold Newkirk electron density model of the ambient corona[6] (Supplementary Figure 10). Considering the close timing between the EUV wave and the Type II shock, We suggest that the EUV wave steeped into a shock at 13:42 UT, which was continuously driven by the MFR but decelerated to 550 km s^{-1}, as measured for the interplanetary shock at 1 AU three days later (Figure 4). The much slower wavefront speed measured in EUV than that of the radio Type II indicates severe projection effects, i.e., the wavefront propagated with a significant velocity component towards the observer.

Supplementary References

[1] Liu, R. *et al.* Structure, Stability, and Evolution of Magnetic Flux Ropes from the Perspective of Magnetic Twist. *Astrophys. J.* **818**, 148 (2016).

[2] Hu, Q., Smith, C. W., Ness, N. F. & Skoug, R. M. Multiple flux rope magnetic ejecta in the solar wind. *Journal of Geophysical Research (Space Physics)* **109**, A03102 (2004).

[3] Liu, R. *et al.* Slow Rise and Partial Eruption of a Double-decker Filament. I. Observations and Interpretation. *Astrophys. J.* **756**, 59 (2012).

[4] Kliem, B. *et al.* Slow Rise and Partial Eruption of a Double-decker Filament. II. A Double Flux Rope Model. *Astrophys. J.* **792**, 107 (2014).

[5] Zurbuchen, T. H. & Richardson, I. G. In-Situ Solar Wind and Magnetic Field Signatures of Interplanetary Coronal Mass Ejections. *Space Sci. Rev.* **123**, 31–43 (2006).

[6] Newkirk, G., Jr. The Solar Corona in Active Regions and the Thermal Origin of the Slowly Varying Component of Solar Radio Radiation. *Astrophys. J.* **133**, 983 (1961).
