# Supplementary material for: Buildup of a highly twisted magnetic flux rope during a solar eruption
Source: Nat Commun. 2017 Nov 6;8:1330. doi: 10.1038/s41467-017-01207-x (PMC5673903; doi:10.1038/s41467-017-01207-x)
Supplement: Supplementary file 3 — Description of Additional Supplementary Files [file 41467_2017_1207_MOESM3_ESM.pdf]

Description of Additional Supplementary Files

File Name: Supplementary Movie 1
Description: Observation of the flare on 4 November 2015 by SDO/AIA in three passbands, 1600, 304, and 131 Å.

File Name: Supplementary Movie 2
Description: Observation of the flare on 4 November 2015 by SDO/AIA in all of the UV and EUV passbands available.

File Name: Supplementary Movie 3
Description: Animation to highlight the dynamic evolution of flare ribbons on the chromosphere in relation to the eruptive structure formed in the corona. The left panel shows a composite of AIA 1600 and 131 Å images, and the right panel a composite of difference images in three AIA EUV passbands, 171, 211, and 131 Å.

File Name: Supplementary Movie 4
Description: Animation to demonstrate how the brightened pixels are identified in 1600 Å and dimmed pixels in 304 and 335 Å.

File Name: Supplementary Movie 5
Description: Data-driven simulation of the coronal magnetic field starting from 13:00 UT on 3 November 2015 until 01:00 UT on 5 November 2015. The left panels shows magnetic field lines traced from footpoints evenly distributed at the photospheric B_z map. The right panel shows the current density distribution in the yz cross section in the center of the computation domain.
